# 10-Methyl-9-phenoxy­carbonyl­acridinium trifluoro­methane­sulfonate monohydrate

**DOI:** 10.1107/S1600536810009979

**Published:** 2010-03-24

**Authors:** Damian Trzybiński, Karol Krzymiński, Artur Sikorski, Jerzy Błażejowski

**Affiliations:** a Faculty of Chemistry, University of Gdańsk, J. Sobieskiego 18, 80-952 Gdańsk, Poland

## Abstract

In the crystal structure of the title compound, C_21_H_16_NO_2_^+^·CF_3_SO_3_^−^·H_2_O, the anions and the water mol­ecules are linked by O—H⋯O inter­actions, while the cations form inversion dimers through π–π inter­actions between acridine ring systems. These dimers are linked by C—H⋯O and C—F⋯π inter­actions to adjacent anions, and by C—H⋯π inter­actions to neighboring cations. The water mol­ecule links two H atoms of the cation by C—H⋯O inter­actions and two adjacent anions by O—H⋯O inter­actions. The acridine and benzene ring systems are oriented at 15.6 (1)°. The carboxyl group is twisted at an angle of 77.0 (1)° relative to the acridine skeleton. The mean planes of the adjacent acridine units are either parallel or inclined at an angle of 18.4 (1)°.

## Related literature

For background to the chemiluminogenic properties of 9-phenoxy­carbonyl-10-methyl­acridinium trifluoro­meth­ane­sulf­on­ates, see: Brown *et al.* (2009 ▶); Rak *et al.* (1999 ▶); Roda *et al.* (2003 ▶); Zomer & Jacquemijns (2001 ▶). For related structures, see: Sikorski *et al.* (2007 ▶); Trzybiński *et al.* (2009 ▶). For inter­molecular inter­actions, see: Bianchi *et al.* (2004 ▶); Dorn *et al.* (2005 ▶); Hunter *et al.* (2001 ▶); Novoa *et al.* (2006 ▶); Takahashi *et al.* (2001 ▶). For the synthesis, see: Sato (1996 ▶); Trzybiński *et al.* (2009 ▶).
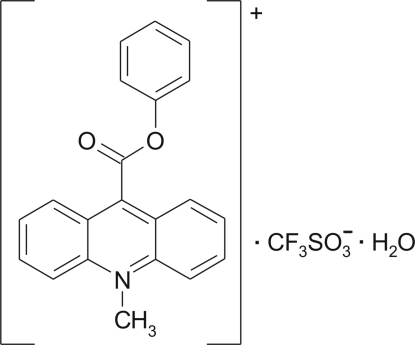


## Experimental

### 

#### Crystal data

C_21_H_16_NO_2_^+^·CF_3_SO_3_^−^·H_2_O
*M* *_r_* = 481.44
Monoclinic, 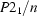

*a* = 11.3807 (4) Å
*b* = 9.5785 (2) Å
*c* = 19.7134 (6) Å
β = 98.172 (3)°
*V* = 2127.14 (11) Å^3^
*Z* = 4
Mo *K*α radiation
μ = 0.22 mm^−1^
*T* = 295 K
0.78 × 0.16 × 0.10 mm

#### Data collection

Oxford Diffraction Gemini R Ultra Ruby CCD diffractometer
Absorption correction: multi-scan (*CrysAlis RED*; Oxford Diffraction, 2008 ▶). *T*_min_ = 0.741, *T*_max_ = 1.000
46462 measured reflections
3792 independent reflections
2422 reflections with *I* > 2σ(*I*)
*R*_int_ = 0.060

#### Refinement

*R*[*F* ^2^ > 2σ(*F* ^2^)] = 0.064
*wR*(*F* ^2^) = 0.211
*S* = 1.03
3792 reflections
305 parameters
3 restraints
H atoms treated by a mixture of independent and constrained refinement
Δρ_max_ = 0.59 e Å^−3^
Δρ_min_ = −0.39 e Å^−3^

### 

Data collection: *CrysAlis CCD* (Oxford Diffraction, 2008 ▶); cell refinement: *CrysAlis RED* (Oxford Diffraction, 2008 ▶); data reduction: *CrysAlis RED*; program(s) used to solve structure: *SHELXS97* (Sheldrick, 2008 ▶); program(s) used to refine structure: *SHELXL97* (Sheldrick, 2008 ▶); molecular graphics: *ORTEP-3* (Farrugia, 1997 ▶); software used to prepare material for publication: *SHELXL97* and *PLATON* (Spek, 2009 ▶).

## Supplementary Material

Crystal structure: contains datablocks global, I. DOI: 10.1107/S1600536810009979/ng2742sup1.cif

Structure factors: contains datablocks I. DOI: 10.1107/S1600536810009979/ng2742Isup2.hkl

Additional supplementary materials:  crystallographic information; 3D view; checkCIF report

## Figures and Tables

**Table 1:** Hydrogen-bond geometry (Å, °) *Cg*4 is the centroid of the C18–C23 ring.

*D*—H⋯*A*	*D*—H	H⋯*A*	*D*⋯*A*	*D*—H⋯*A*
C2—H2⋯O28^i^	0.93	2.59	3.424 (4)	149
C8—H8⋯O25	0.93	2.52	3.360 (6)	150
C19—H19⋯O25	0.93	2.57	3.232 (5)	129
C24—H24*A*⋯*Cg*4^ii^	0.96	2.69	3.484 (4)	140
C24—H24*C*⋯O29^ii^	0.96	2.60	3.544 (5)	168
O25—H25*A*⋯O27	0.85 (4)	1.98 (3)	2.816 (5)	170 (8)
O25—H25*B*⋯O28^iii^	0.86 (4)	2.14 (6)	2.948 (6)	156 (7)

Symmetry codes: (i) 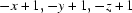; (ii) 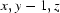; (iii) 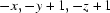.

**Table 2:** C—F⋯π inter­actions (Å, °) *Cg*1 and *Cg*2 are the centroids of the C9/N10/C11–C14 and C1–C4/C11/C12 rings, respectively.

*X*—*I*⋯*J*	*I*⋯*J*	*X*⋯*J*	*X*—*I*⋯*J*
C30—F31⋯*Cg*2^iv^	3.269 (3)	4.075 (4)	117.8 (2)
C30—F32⋯*Cg*1^iv^	3.744 (3)	4.463 (4)	116.1 (3)

Symmetry code: (iv) 
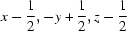
.

**Table 3:** π–π inter­actions (Å, °) *Cg*1 and *Cg*2 are the centroids of the C9/N10/C11–C14 and C1–C4/C11/C12 rings, respectively. *CgI*⋯*CgJ* is the distance between ring centroids. The dihedral angle is that between the planes of the rings *I* and *J. CgI*_Perp is the perpendicular distance of *CgI* from ring *J. CgI*_Offset is the distance between *CgI* and the perpendicular projection of *CgJ* on ring *I*.

*I*	*J*	*CgI*⋯*CgJ*	Dihedral angle	*CgI*_Perp	*CgI*_Offset
1	2^v^	3.682 (2)	1.92 (1)	3.568 (1)	0.909 (1)
2	1^v^	3.682 (2)	1.92 (1)	3.591 (1)	0.814 (1)

Symmetry code: (v) −*x* + 1, −*y*, −*z* + 1.

**Fig. 1.**
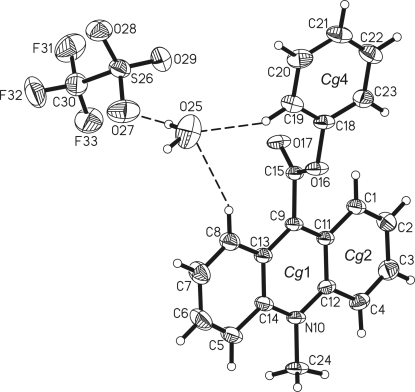
The molecular structure of the title compound showing the atom labeling scheme. Displacement ellipsoids are drawn at the 25% probability level and H atoms are shown as small spheres of arbitrary radius. Cg1, Cg2 and Cg4 denote the ring centroids. The O–H···O and C–H···O hydrogen bonds are represented by dashed lines.

**Fig. 2.**
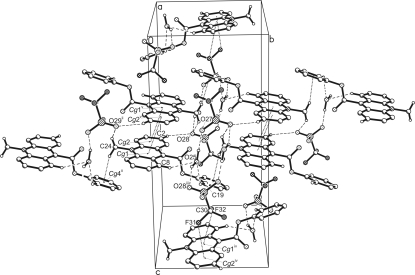
The arrangement of the ions in the crystal structure. The O–H···O and C–H···O interactions are represented by dashed lines, the C–H···π, C–F···π and π–π contacts by dotted lines. H atoms not involved in interactions have been omitted. [Symmetry codes: (i) –x + 1, –y + 1, –z + 1; (ii) x, y – 1, z; (iii) –x, –y + 1, –z + 1; (iv) x – 1/2, –y + 1/2, z – 1/2; (v) –x + 1, –y, –z + 1.]

## supplementary crystallographic information

### Comment

The crystal structures of six 9-phenoxycarbonyl-10-methylacridinium trifluoromethanesulfonates can be found in the Cambridge Structural Database. All of them were determined in our laboratory and concern derivatives substituted in the phenyl fragment. For a long time we were unable to obtain crystals of the parent compound, i.e. unsubstituted 9-phenoxycarbonyl-10-methylacridinium trifluoromethanesulfonate, suitable for X-Ray investigations. Eventually we succeeded, and we present here the crystal structure of the monohydrate of this compound. The reason for our interest in this group of compounds is their chemiluminogenic properties, which means they can be used as chemiluminescent indicators or the chemiluminogenic fragments of chemiluminescent labels (Zomer & Jacquemijns, 2001). These compounds are rouitenely applied in assays of biologically and environmentally important entities such as antigens, antibodies, enzymes or DNA fragments (Roda *et al.*, 2003; Brown *et al.*, 2009). The cations of the above mentioned salts undergo oxidation with hydrogen peroxide in alkaline media; at the same time the phenoxycarbonyl fragment is removed and the remainder of the molecule is converted to electronically excited, light-emitting 10-methyl-9-acridinone (Rak *et al.*, 1999). This forms the basis for analytical applications (Zomer & Jacquemijns, 2001).

In the cation of the title compound (Fig. 1), the bond lengths and angles characterizing the geometry of the acridinium moiety are typical of acridine-based derivatives (Sikorski *et al.*, 2007; Trzybiński *et al.*, 2009). With respective average deviations from planarity of 0.0292 (3) Å and 0.0016 (3) Å, the acridine and benzene ring systems are oriented at 15.6 (1)°. The carboxyl group is twisted at an angle of 77.0 (1)° relative to the acridine skeleton. The mean planes of the adjacent acridine moieties are parallel (at an angle of 0.0 (1)°) or inclined at an angle of 18.4 (1)° in the lattice.

In the crystal structure, the anions form hydrates with water molecules through O–H···O interactions, while the inversely oriented cations form dimers through π–π interactions involving acridine moieties (Tables 1 and 3, Figs. 1 and 2). These dimers are linked by C–H···O (Table 1, Fig. 2) and C–F···π (Table 2, Fig. 2) interactions to adjacent anions, and by C–H···π (Table 1, Fig. 2) interactions to neighboring cations. The water molecule links two sites of the cation by C–H···O interactions and two adjacent anions by O–H···O interactions (Table 1, Figs. 1 and 2). The O–H···O and C–H···O interactions are of the hydrogen bond type (Bianchi *et al.*, 2004; Novoa *et al.*, 2006). The C–H···π interactions should be of an attractive nature (Takahashi *et al.*, 2001), like the C–F···π (Dorn *et al.*, 2005) and the π–π (Hunter *et al.*, 2001) interactions. The crystal structure is stabilized by a network of these short-range specific interactions and by long-range electrostatic interactions between ions.

### Experimental

The compound was synthesized following a procedure described elsewhere (Trzybiński *et al.*, 2009). 9-(Chlorocarbonyl)-acridine was prepared by treating acridine-9-carboxylic acid with a tenfold molar excess of thionyl chloride. The compound obtained was esterified with phenol in anhydrous dichloromethane in the presence of *N*,*N*-diethylethanamine and a catalytic amount of *N*,*N*-dimethyl-4-pyridinamine (room temperature, 15h). The product – phenyl acridine-9-carboxylate – was purified chromatographically (SiO_2_, cyclohexane/ethyl acetate, 3/2 v/v) and quaternarized with a five-fold molar excess of methyl trifluoromethanesulfonate dissolved in anhydrous dichloromethane (under an Ar atmosphere at room temperature for 3h) (Sato, 1996). The crude 9-phenoxycarbonyl-10-methylacridinium trifluoromethanesulfonate was dissolved in a small amount of ethanol, filtered and precipitated with a 25 v/v excess of diethyl ether. Yellow crystals suitable for X-ray investigations were grown from ethanol/H_2_O, 4/1 v/v, solution (m.p. 263-265K).

### Refinement

The H-atoms of the water molecule were located on a Fourier-difference map, restrained by DFIX command 0.85 for O–H distances and by DFIX 1.39 for H···H distance and refined as riding with *U*_iso_(H) = 1.5U_eq_(O). All other H atoms were positioned geometrically, with C—H = 0.93 Å and 0.96 Å for the aromatic and methyl H atoms, respectively, and constrained to ride on their parent atoms with *U*_iso_(H) = *xU*_eq_(C), where *x* = 1.2 for the aromatic H atoms and *x* = 1.5 for the methyl H atoms.

### Crystal data

**Table:**

C_21_H_16_NO_2_^+^·CF_3_SO_3_^−^·H_2_O	*F*(000) = 992
*M**_r_* = 481.44	*D*_x_ = 1.503 Mg m^−^^3^
Monoclinic, *P*2_1_/*n*	Mo *K*α radiation, λ = 0.71073 Å
Hall symbol: -P 2yn	Cell parameters from 15973 reflections
*a* = 11.3807 (4) Å	θ = 3.0–29.3°
*b* = 9.5785 (2) Å	µ = 0.22 mm^−^^1^
*c* = 19.7134 (6) Å	*T* = 295 K
β = 98.172 (3)°	Needle, yellow
*V* = 2127.14 (11) Å^3^	0.78 × 0.16 × 0.10 mm
*Z* = 4	

### Data collection

**Table:**

Oxford Diffraction Gemini R Ultra Ruby CCD diffractometer	3792 independent reflections
Radiation source: Enhanced (Mo) X-ray Source	2422 reflections with *I* > 2σ(*I*)
graphite	*R*_int_ = 0.060
Detector resolution: 10.4002 pixels mm^-1^	θ_max_ = 25.1°, θ_min_ = 3.0°
ω scans	*h* = −13→13
Absorption correction: multi-scan (*CrysAlis RED*; Oxford Diffraction, 2008).	*k* = −11→11
*T*_min_ = 0.741, *T*_max_ = 1.000	*l* = −23→23
46462 measured reflections	

### Refinement

**Table:**

Refinement on *F*^2^	Primary atom site location: structure-invariant direct methods
Least-squares matrix: full	Secondary atom site location: difference Fourier map
*R*[*F*^2^ > 2σ(*F*^2^)] = 0.064	Hydrogen site location: inferred from neighbouring sites
*wR*(*F*^2^) = 0.211	H atoms treated by a mixture of independent and constrained refinement
*S* = 1.03	*w* = 1/[σ^2^(*F*_o_^2^) + (0.146*P*)^2^] where *P* = (*F*_o_^2^ + 2*F*_c_^2^)/3
3792 reflections	(Δ/σ)_max_ < 0.001
305 parameters	Δρ_max_ = 0.59 e Å^−^^3^
3 restraints	Δρ_min_ = −0.39 e Å^−^^3^

### Special details

**Table:**

Geometry. All esds (except the esd in the dihedral angle between two l.s. planes) are estimated using the full covariance matrix. The cell esds are taken into account individually in the estimation of esds in distances, angles and torsion angles; correlations between esds in cell parameters are only used when they are defined by crystal symmetry. An approximate (isotropic) treatment of cell esds is used for estimating esds involving l.s. planes.

### Fractional atomic coordinates and isotropic or equivalent isotropic displacement parameters (Å^2^)

**Table:**

	*x*	*y*	*z*	*U*_iso_*/*U*_eq_	
C1	0.6362 (3)	0.0857 (3)	0.60005 (15)	0.0534 (7)	
H1	0.6328	0.1816	0.6067	0.064*	
C2	0.7417 (3)	0.0248 (3)	0.59672 (17)	0.0647 (8)	
H2	0.8103	0.0788	0.5999	0.078*	
C3	0.7481 (3)	−0.1205 (4)	0.58836 (19)	0.0703 (9)	
H3	0.8218	−0.1620	0.5877	0.084*	
C4	0.6494 (3)	−0.2020 (3)	0.58122 (16)	0.0613 (8)	
H4	0.6561	−0.2979	0.5753	0.074*	
C5	0.2201 (3)	−0.2410 (3)	0.56540 (18)	0.0700 (10)	
H5	0.2234	−0.3363	0.5567	0.084*	
C6	0.1149 (4)	−0.1788 (4)	0.5680 (2)	0.0859 (12)	
H6	0.0464	−0.2332	0.5615	0.103*	
C7	0.1041 (3)	−0.0366 (4)	0.5799 (2)	0.0822 (11)	
H7	0.0296	0.0029	0.5805	0.099*	
C8	0.2025 (3)	0.0440 (3)	0.59056 (17)	0.0637 (9)	
H8	0.1954	0.1389	0.5991	0.076*	
C9	0.4195 (3)	0.0648 (2)	0.59775 (13)	0.0450 (7)	
N10	0.4351 (2)	−0.2193 (2)	0.57446 (11)	0.0476 (6)	
C11	0.5304 (3)	0.0064 (3)	0.59353 (12)	0.0439 (7)	
C12	0.5374 (3)	−0.1418 (3)	0.58269 (13)	0.0468 (7)	
C13	0.3168 (3)	−0.0151 (3)	0.58886 (14)	0.0493 (7)	
C14	0.3255 (3)	−0.1614 (3)	0.57599 (14)	0.0506 (7)	
C15	0.4143 (2)	0.2199 (3)	0.61254 (14)	0.0454 (7)	
O16	0.4005 (2)	0.24024 (17)	0.67778 (10)	0.0597 (6)	
O17	0.4242 (2)	0.30844 (19)	0.57195 (10)	0.0647 (6)	
C18	0.4040 (3)	0.3808 (3)	0.70187 (14)	0.0523 (8)	
C19	0.3003 (3)	0.4502 (3)	0.70065 (17)	0.0635 (9)	
H19	0.2286	0.4080	0.6836	0.076*	
C20	0.3034 (4)	0.5869 (4)	0.7257 (2)	0.0763 (11)	
H20	0.2335	0.6375	0.7252	0.092*	
C21	0.4108 (4)	0.6459 (4)	0.7509 (2)	0.0797 (11)	
H21	0.4134	0.7366	0.7679	0.096*	
C22	0.5133 (4)	0.5729 (4)	0.7514 (2)	0.0815 (11)	
H22	0.5855	0.6142	0.7683	0.098*	
C23	0.5108 (3)	0.4375 (3)	0.72675 (17)	0.0669 (9)	
H23	0.5805	0.3866	0.7272	0.080*	
C24	0.4419 (3)	−0.3729 (3)	0.56256 (17)	0.0637 (9)	
H24A	0.3966	−0.4213	0.5928	0.095*	
H24B	0.5232	−0.4026	0.5714	0.095*	
H24C	0.4102	−0.3937	0.5159	0.095*	
O25	0.1330 (4)	0.3806 (6)	0.5581 (2)	0.1480 (15)	
H25A	0.136 (7)	0.397 (9)	0.5162 (16)	0.222*	
H25B	0.064 (4)	0.350 (10)	0.563 (4)	0.222*	
S26	0.15677 (9)	0.55386 (9)	0.38344 (5)	0.0715 (4)	
O27	0.1121 (4)	0.4342 (3)	0.41645 (19)	0.1242 (12)	
O28	0.0899 (3)	0.6777 (3)	0.38719 (15)	0.0997 (9)	
O29	0.2829 (3)	0.5641 (3)	0.39743 (18)	0.1067 (10)	
C30	0.1269 (4)	0.5037 (5)	0.2942 (2)	0.0903 (12)	
F31	0.1687 (3)	0.6041 (3)	0.25626 (16)	0.1375 (12)	
F32	0.0152 (3)	0.4950 (4)	0.27144 (17)	0.1380 (12)	
F33	0.1787 (3)	0.3873 (3)	0.28194 (15)	0.1227 (10)	

### Atomic displacement parameters (Å^2^)

**Table:**

	*U*^11^	*U*^22^	*U*^33^	*U*^12^	*U*^13^	*U*^23^
C1	0.065 (2)	0.0385 (14)	0.0567 (17)	−0.0056 (13)	0.0088 (14)	−0.0035 (12)
C2	0.061 (2)	0.0628 (19)	0.071 (2)	−0.0063 (16)	0.0092 (16)	−0.0077 (15)
C3	0.067 (2)	0.068 (2)	0.076 (2)	0.0102 (18)	0.0113 (17)	−0.0048 (17)
C4	0.078 (2)	0.0434 (16)	0.063 (2)	0.0136 (15)	0.0108 (16)	−0.0023 (13)
C5	0.082 (3)	0.0491 (17)	0.078 (2)	−0.0199 (17)	0.0084 (18)	−0.0067 (15)
C6	0.063 (3)	0.082 (3)	0.111 (3)	−0.027 (2)	0.008 (2)	−0.018 (2)
C7	0.056 (2)	0.086 (3)	0.104 (3)	−0.0065 (19)	0.011 (2)	−0.019 (2)
C8	0.064 (2)	0.0529 (17)	0.074 (2)	−0.0019 (15)	0.0097 (17)	−0.0084 (14)
C9	0.0643 (19)	0.0295 (13)	0.0413 (14)	−0.0039 (12)	0.0074 (13)	−0.0014 (10)
N10	0.0708 (17)	0.0262 (10)	0.0461 (13)	−0.0039 (10)	0.0091 (11)	−0.0018 (8)
C11	0.0603 (18)	0.0320 (13)	0.0392 (14)	−0.0017 (12)	0.0065 (12)	−0.0017 (10)
C12	0.0670 (19)	0.0334 (13)	0.0398 (14)	0.0012 (13)	0.0072 (13)	0.0018 (10)
C13	0.0632 (19)	0.0371 (14)	0.0469 (15)	−0.0020 (13)	0.0058 (13)	−0.0028 (11)
C14	0.066 (2)	0.0377 (14)	0.0481 (16)	−0.0117 (13)	0.0071 (13)	−0.0022 (11)
C15	0.0543 (18)	0.0311 (13)	0.0505 (17)	−0.0011 (11)	0.0071 (13)	−0.0024 (12)
O16	0.0985 (17)	0.0294 (9)	0.0546 (13)	−0.0024 (9)	0.0226 (11)	−0.0024 (8)
O17	0.1108 (19)	0.0318 (10)	0.0544 (12)	−0.0024 (10)	0.0214 (12)	0.0035 (9)
C18	0.082 (2)	0.0329 (14)	0.0457 (15)	−0.0025 (14)	0.0201 (15)	−0.0031 (11)
C19	0.072 (2)	0.0525 (18)	0.069 (2)	−0.0098 (16)	0.0234 (17)	−0.0085 (14)
C20	0.090 (3)	0.0555 (19)	0.091 (3)	0.0138 (18)	0.039 (2)	−0.0100 (17)
C21	0.111 (3)	0.0485 (18)	0.083 (3)	−0.011 (2)	0.026 (2)	−0.0207 (17)
C22	0.099 (3)	0.064 (2)	0.080 (3)	−0.017 (2)	0.010 (2)	−0.0235 (18)
C23	0.077 (2)	0.0539 (18)	0.069 (2)	0.0022 (16)	0.0076 (17)	−0.0084 (15)
C24	0.096 (2)	0.0253 (13)	0.072 (2)	−0.0041 (14)	0.0191 (17)	−0.0056 (12)
O25	0.121 (3)	0.191 (4)	0.130 (3)	0.034 (3)	0.009 (2)	0.014 (3)
S26	0.0867 (8)	0.0584 (5)	0.0688 (6)	−0.0088 (4)	0.0084 (5)	−0.0088 (4)
O27	0.174 (3)	0.092 (2)	0.118 (3)	−0.020 (2)	0.058 (2)	0.0251 (17)
O28	0.133 (3)	0.0679 (16)	0.098 (2)	0.0144 (16)	0.0154 (17)	−0.0183 (14)
O29	0.090 (2)	0.100 (2)	0.119 (2)	−0.0072 (16)	−0.0236 (17)	−0.0098 (17)
C30	0.094 (3)	0.081 (3)	0.092 (3)	0.014 (2)	0.001 (2)	−0.019 (2)
F31	0.192 (3)	0.131 (2)	0.0935 (19)	0.021 (2)	0.034 (2)	0.0265 (16)
F32	0.105 (2)	0.155 (3)	0.138 (2)	0.0063 (18)	−0.0372 (18)	−0.058 (2)
F33	0.161 (3)	0.0951 (18)	0.110 (2)	0.0314 (17)	0.0119 (17)	−0.0364 (14)

### Geometric parameters (Å, °)

**Table:**

C1—C2	1.345 (4)	C15—O17	1.182 (3)
C1—C11	1.414 (4)	C15—O16	1.332 (3)
C1—H1	0.9300	O16—C18	1.426 (3)
C2—C3	1.405 (5)	C18—C19	1.352 (5)
C2—H2	0.9300	C18—C23	1.357 (5)
C3—C4	1.358 (5)	C19—C20	1.398 (5)
C3—H3	0.9300	C19—H19	0.9300
C4—C12	1.404 (4)	C20—C21	1.374 (6)
C4—H4	0.9300	C20—H20	0.9300
C5—C6	1.345 (5)	C21—C22	1.358 (6)
C5—C14	1.412 (4)	C21—H21	0.9300
C5—H5	0.9300	C22—C23	1.384 (5)
C6—C7	1.391 (5)	C22—H22	0.9300
C6—H6	0.9300	C23—H23	0.9300
C7—C8	1.351 (5)	C24—H24A	0.9600
C7—H7	0.9300	C24—H24B	0.9600
C8—C13	1.424 (4)	C24—H24C	0.9600
C8—H8	0.9300	O25—H25A	0.85 (2)
C9—C13	1.387 (4)	O25—H25B	0.85 (2)
C9—C11	1.393 (4)	S26—O28	1.417 (3)
C9—C15	1.517 (3)	S26—O29	1.426 (3)
N10—C14	1.369 (4)	S26—O27	1.445 (3)
N10—C12	1.371 (4)	S26—C30	1.809 (5)
N10—C24	1.494 (3)	C30—F32	1.290 (5)
C11—C12	1.439 (4)	C30—F33	1.299 (5)
C13—C14	1.430 (4)	C30—F31	1.346 (5)
			
C2—C1—C11	121.2 (3)	O17—C15—O16	125.8 (2)
C2—C1—H1	119.4	O17—C15—C9	124.2 (2)
C11—C1—H1	119.4	O16—C15—C9	110.0 (2)
C1—C2—C3	119.8 (3)	C15—O16—C18	117.2 (2)
C1—C2—H2	120.1	C19—C18—C23	123.0 (3)
C3—C2—H2	120.1	C19—C18—O16	118.4 (3)
C4—C3—C2	121.8 (3)	C23—C18—O16	118.6 (3)
C4—C3—H3	119.1	C18—C19—C20	118.4 (3)
C2—C3—H3	119.1	C18—C19—H19	120.8
C3—C4—C12	120.1 (3)	C20—C19—H19	120.8
C3—C4—H4	120.0	C21—C20—C19	119.3 (3)
C12—C4—H4	120.0	C21—C20—H20	120.3
C6—C5—C14	119.8 (3)	C19—C20—H20	120.3
C6—C5—H5	120.1	C22—C21—C20	120.6 (3)
C14—C5—H5	120.1	C22—C21—H21	119.7
C5—C6—C7	122.7 (3)	C20—C21—H21	119.7
C5—C6—H6	118.6	C21—C22—C23	120.3 (4)
C7—C6—H6	118.6	C21—C22—H22	119.8
C8—C7—C6	119.7 (4)	C23—C22—H22	119.8
C8—C7—H7	120.2	C18—C23—C22	118.3 (4)
C6—C7—H7	120.2	C18—C23—H23	120.8
C7—C8—C13	120.6 (3)	C22—C23—H23	120.8
C7—C8—H8	119.7	N10—C24—H24A	109.5
C13—C8—H8	119.7	N10—C24—H24B	109.5
C13—C9—C11	121.7 (2)	H24A—C24—H24B	109.5
C13—C9—C15	120.6 (3)	N10—C24—H24C	109.5
C11—C9—C15	117.7 (2)	H24A—C24—H24C	109.5
C14—N10—C12	122.6 (2)	H24B—C24—H24C	109.5
C14—N10—C24	118.1 (2)	H25A—O25—H25B	110 (3)
C12—N10—C24	119.3 (2)	O28—S26—O29	117.75 (19)
C9—C11—C1	123.1 (2)	O28—S26—O27	114.5 (2)
C9—C11—C12	118.3 (2)	O29—S26—O27	112.1 (2)
C1—C11—C12	118.6 (3)	O28—S26—C30	104.19 (19)
N10—C12—C4	122.2 (2)	O29—S26—C30	104.5 (2)
N10—C12—C11	119.2 (3)	O27—S26—C30	101.4 (2)
C4—C12—C11	118.6 (3)	F32—C30—F33	109.3 (4)
C9—C13—C8	122.3 (2)	F32—C30—F31	105.2 (4)
C9—C13—C14	119.0 (3)	F33—C30—F31	107.7 (4)
C8—C13—C14	118.7 (3)	F32—C30—S26	113.3 (3)
N10—C14—C5	122.4 (3)	F33—C30—S26	112.4 (3)
N10—C14—C13	119.2 (2)	F31—C30—S26	108.6 (3)
C5—C14—C13	118.5 (3)		
			
C11—C1—C2—C3	−1.6 (5)	C6—C5—C14—N10	179.5 (3)
C1—C2—C3—C4	2.2 (5)	C6—C5—C14—C13	−0.4 (5)
C2—C3—C4—C12	−0.6 (5)	C9—C13—C14—N10	1.6 (4)
C14—C5—C6—C7	0.9 (6)	C8—C13—C14—N10	−179.8 (3)
C5—C6—C7—C8	−1.1 (7)	C9—C13—C14—C5	−178.5 (3)
C6—C7—C8—C13	0.8 (6)	C8—C13—C14—C5	0.1 (4)
C13—C9—C11—C1	177.8 (3)	C13—C9—C15—O17	−104.9 (4)
C15—C9—C11—C1	−2.2 (4)	C11—C9—C15—O17	75.1 (4)
C13—C9—C11—C12	−2.8 (4)	C13—C9—C15—O16	76.8 (3)
C15—C9—C11—C12	177.2 (2)	C11—C9—C15—O16	−103.2 (3)
C2—C1—C11—C9	178.9 (3)	O17—C15—O16—C18	−3.4 (4)
C2—C1—C11—C12	−0.4 (4)	C9—C15—O16—C18	174.8 (2)
C14—N10—C12—C4	179.8 (3)	C15—O16—C18—C19	94.4 (3)
C24—N10—C12—C4	−0.8 (4)	C15—O16—C18—C23	−87.5 (3)
C14—N10—C12—C11	0.1 (4)	C23—C18—C19—C20	0.5 (5)
C24—N10—C12—C11	179.5 (2)	O16—C18—C19—C20	178.6 (3)
C3—C4—C12—N10	178.8 (3)	C18—C19—C20—C21	−0.4 (5)
C3—C4—C12—C11	−1.5 (4)	C19—C20—C21—C22	0.4 (6)
C9—C11—C12—N10	2.3 (3)	C20—C21—C22—C23	−0.4 (6)
C1—C11—C12—N10	−178.3 (2)	C19—C18—C23—C22	−0.6 (5)
C9—C11—C12—C4	−177.4 (2)	O16—C18—C23—C22	−178.7 (3)
C1—C11—C12—C4	2.0 (4)	C21—C22—C23—C18	0.5 (6)
C11—C9—C13—C8	−177.7 (3)	O28—S26—C30—F32	−53.1 (4)
C15—C9—C13—C8	2.3 (4)	O29—S26—C30—F32	−177.2 (3)
C11—C9—C13—C14	0.9 (4)	O27—S26—C30—F32	66.1 (4)
C15—C9—C13—C14	−179.1 (2)	O28—S26—C30—F33	−177.6 (3)
C7—C8—C13—C9	178.3 (3)	O29—S26—C30—F33	58.2 (4)
C7—C8—C13—C14	−0.3 (5)	O27—S26—C30—F33	−58.5 (4)
C12—N10—C14—C5	178.0 (3)	O28—S26—C30—F31	63.3 (4)
C24—N10—C14—C5	−1.3 (4)	O29—S26—C30—F31	−60.8 (3)
C12—N10—C14—C13	−2.1 (4)	O27—S26—C30—F31	−177.5 (3)
C24—N10—C14—C13	178.6 (2)		

### Hydrogen-bond geometry (Å, °)

**Table:**

Cg4 is the centroid of the C18–C23 ring.

**Table:**

*D*—H···*A*	*D*—H	H···*A*	*D*···*A*	*D*—H···*A*
C2—H2···O28^i^	0.93	2.59	3.424 (4)	149
C8—H8···O25	0.93	2.52	3.360 (6)	150
C19—H19···O25	0.93	2.57	3.232 (5)	129
C24—H24*A*···*Cg*4^ii^	0.96	2.69	3.484 (4)	140
C24—H24*C*···O29^ii^	0.96	2.60	3.544 (5)	168
O25—H25*A*···O27	0.85 (4)	1.98 (3)	2.816 (5)	170 (8)
O25—H25*B*···O28^iii^	0.86 (4)	2.14 (6)	2.948 (6)	156 (7)

Symmetry codes:  (i) −*x*+1, −*y*+1, −*z*+1; (ii) *x*, *y*−1, *z*; (iii) −*x*, −*y*+1, −*z*+1.

### Table 2 C—F···π interactions (Å, °)

*Cg*1 and *Cg*2 are the centroids of the C9/N10/C11–C14 and C1–C4/C11/C12 rings, respectively.

**Table:**

*X*—*I*···*J*	*I*···*J*	*X*···*J*	*X*—*I*···*J*
C30—F31···*Cg*2^iv^	3.269 (3)	4.075 (4)	117.8 (2)
C30—F32···*Cg*1^iv^	3.744 (3)	4.463 (4)	116.1 (3)

Symmetry code: (iv) x-1/2, -y+1/2, z-1/2.

### Table 3 π–π interactions (Å, °)

*Cg*1 and *Cg*2 are the centroids of the C9/N10/C11–C14 and C1–C4/C11/C12 rings, respectively. Cg*I*···Cg*J* is the distance between ring centroids. The dihedral angle is that between the planes of the rings *I* and *J*. *CgI*_Perp is the perpendicular distance of *CgI* from ring *J*. Cg*I*_Offset is the distance between *CgI* and the perpendicular projection of *CgJ* on ring *I*.

**Table:**

*I*	*J*	*CgI*···*CgJ*	Dihedral angle	*CgI*_Perp	*CgI*_Offset
1	2^v^	3.682 (2)	1.92 (1)	3.568 (1)	0.909 (1)
2	1^v^	3.682 (2)	1.92 (1)	3.591 (1)	0.814 (1)

Symmetry code: (v) -x+1, -y, -z+1.
